# Death and *best* interests: a response to the legal challenge

**DOI:** 10.1258/ce.2010.010033

**Published:** 2010-12

**Authors:** Paul Baines

**Affiliations:** Wellcome Trust Biomedical Ethics Research Fellow, Centre for Professional Ethics, Room CBC 2.001, Chancellor's Building, Keele University, Staffordshire ST5 5BG, UK

## Abstract

In an earlier paper I argued that we do not have an objective conception of best interests and that this is a particular problem because the courts describe that they use an ‘…objective approach or test. That test is the best interests of the patient’ when choosing for children. I further argued that there was no obvious way in which we could hope to develop an objective notion of best interests. As well as this, I argued that a best-interest-based approach was a particular problem around the time of death of some children. A response from a legal perspective argued that, while there is not a clear conception of objective best interests, the courts have a well-described approach to finding a child's objective best interests. In this paper, I argue that without clear agreement on an objective conception of best interests, the courts are unable to locate an objective sense of best interests and that the solutions do not solve the problems that were identified in the initial paper ‘Death and *best* interests’.

In an earlier paper ‘Death and *best* interests’^1^, I argued that we do not have an objective notion of best interests, describing this as a serious problem in deciding how medical decisions should be made for critically ill children. Among the responses that could be made to my claims is that the courts are charged with the responsibility of deciding in a child's best interests and are able to do so, regularly making findings of what would be in a child's objective best interests. I will consider the legal challenge to my claims, and am grateful for Bridgeman's clear criticisms^2^ of my paper stating the legal position plainly. Before I do, I should perhaps have been more explicit (as others have been) in stating that my aim was a broader critical analysis of the concept of best interests^3^ especially given my legal example. In my paper, I used *an NHS trust* v *MB* ^4^ only as an example and as a clear statement of the law, in order to seek a broad examination of the notion of best interests. A legal conception of best interests may be different from a philosophical one, which may explain some disagreement. But the notion of best interests used in law should be based on sound principles and subject to evaluation and criticism. My response addresses two points. First, I reiterate and argue for my claim that without a clear objective notion of best interests, the courts are unable to fulfil their stated aim of using an objective test of best interests. There may be a clear legal framework, but this is not an objective framework. Secondly, I argue that Keown's response to Lord Mustill fails to demonstrate that patients in Tony Bland's condition have interests.

## An objective test of best interests

Bridgeman and I agree, albeit for different reasons, that ‘… there is no one objective best interest’.^5^ There are two broad reasons why an individual's best interests may not be able to be found. First, there may be no such thing as objective best interests. In a pluralistic world, there may be entirely different and incommensurable ways in which we could live a good life, and the claim that one life is better than another makes no sense. Even if it is true that in some circumstances lives may be compared one with another and one judged better than another, this may not be true for very different lives in different domains. For example, an individual may have different options open to them to pursue a life as an elite sportsman, a successful businessman or a rural farmer and it may be difficult to choose between them. This is in part because of uncertainty over how the different lives will turn out; however, even if an individual could be assuredly successful in any one of them, there is no clear sense as to which one of those would be (in an objective sense) best, because they are so very different. It is not possible to evaluate which is best in important ways. This is an ontological claim. The other idea is that it may be true that there is such a thing as an individual's objective best interests, but there may be no way that we can ever know what they are (an epistemological problem). Either way, Bridgeman and I agree that there is no one objective best interests.

Bridgeman and I part company in the following paragraph. My claim was ‘I have not argued that we will not be able to develop an objective test of best interests, merely that we have not yet done so, nor does there seem to be a clear way in which we could work towards developing an objective concept of best interests’.^6^ Bridgeman responds ‘It is in the next step of his argument, that I disagree with Baines, who then asserts that there is no “clear framework within which best interests can be assessed”’ (p. 172). While the references offered by him to support this view do illustrate that there is scope for different approaches to be adopted to assessment of best interests, the law has, in recent years, established the principles and approach to be adopted, establishing a very clear framework for assessment'.^7^ It is important to be clear what our disagreement is about. I make the claim that we have no clear way to establish an objective test of best interests. The complete sentence that Bridgeman partially quoted is ‘Furthermore, not only do we not have an objective test of best interests we do not even have a clear framework within which best interests can be assessed’.^8^ Bridgeman disagrees. Our disagreement, then, may take two routes. First, and more easily, in accepting that there are no objective best interests, Bridgeman may claim that the courts have *a* framework for deciding a *legal* conception of interests, but then these are not objective best interests. Remember, Mr Justice Holman declared that ‘My task, difficult enough in itself, is to decide, and only to decide, where the objective balance of best interests of M lies’.^9^ If so, my claim is that Bridgeman should join with me in recognizing the limitations of objectivity and that the claim to objectivity adopted by the courts should be dropped or modified. If, as seems evident, objectivity is sought in the legal process, the legal procedures should be consciously examined and if necessary adapted to make them more objective or to eliminate as many threats to objectivity as may be removed, but, importantly, with the acceptance that these are still not objective best interests. The second and more interesting disagreement is if Bridgeman maintains that there are no objective interests, but that the courts can find a child's objective interests.

If the claim is that there are no objective best interests but the court can identify some objectivity in a child's best interests, this may be through the use of an objective process, approach or test so that we can decide on what we *treat* as a person's objective interests. This approach must be adopted if Bridgeman hopes to retain the claim to objectivity within best interests, but recognizes that there is no such thing as objective best interests. And if we accept this approach, then we must recognize that the idea of best interests as used in law is merely a constructed process. But if this approach is taken, it should be made explicit. Two concerns follow from this. First, although Bridgeman may claim that the courts have a well-established way to decide best interests, there is no general agreement as to what would be in someone's interests; either more broadly in society or within philosophy. Furthermore, there is no agreement on a framework to assess someone's interests. So even if the courts have a way to decide on best interests, as my claim was that there was no framework to approach objective best interests, then the legal conception of best interests should be open to broader scrutiny and criticism to determine whether the legal conception satisfies the criterion of objectivity. Bridgeman criticizes a medical conception of best interests as being limited ‘Best interests is not confined to medical best interests, rather it embraces all welfare issues including the “medical, emotional, sensory (pleasure, pain and suffering) and instinctive (the human instinct to survive)”’.^10^ When considering a child's best interests, doctors do so from a medical perspective, but would strive to give a clear sense of a child's overall best interests, not limited solely to ‘medical’ best interests. The medical perspective may be distorted by the doctors' interaction with, and knowledge of, a child gained only in medical situations, but it is not clear why a legal conception, unless subject to close scrutiny, should be preferred. The doctors and nurses will have interacted with the child in these sorts of cases over a considerable period of time. The judge need not have visited the child at all. And in recognizing that there is no objective notion of best interests it would be wise to consult broadly on which factors should be considered in best interests or what sort of process should be used to determine best interests. As Bridgeman notes, judges themselves will not be objective: ‘Critical legal and feminist scholars have shown that the espoused goal of objectivity is unrealizable, as judges bring their own values, experiences and perspectives to their judging’.^11^

Secondly, it is important to recognize that this approach to best interests is different from at least some of the usual workings of the legal system. When a murderer is convicted, he is convicted because we believe that he is guilty, not merely because he has undergone an objective process and that this was as close to the murderer as we could get. We believe that he is the person who murdered someone and if it becomes clear for whatever reasons, perhaps forensic or gene-matching evidence, that he is not the murderer, then no matter how objective the approach had been, he would be released. The way that Bridgeman uses the idea of best interests is very different. There is no fact or truth that is found by the court. If the courts are to use the objectivity of the approach, and not the (missing) objectivity inherent in the idea that there are objective best interests to ground an objective notion of best interests, then it is important to make this explicit and to review the approach or methodology to ensure that this is objective, and that this is understood by all. It may be important, then, to describe that we have used an ‘objective approach to alight on what we believe someone's interests to be’. This is a mouthful and perhaps in use it would be shortened to ‘objective best interests’, but if challenged we should recognize that the phrase ‘objective best interests’ is shorthand for the longer and more accurate description.

So how could we go about injecting objectivity into the way that we find a person's best interests? In some areas despite a failure to agree on any objective sense of what is correct or what is best, we are able to achieve agreement. For example, we have no objective agreement as to what is best in literature or art, but regardless of this we are able to have contests where works of art or books are assessed and one is recognized as the winner. Perhaps we can look to the way that book or art contests are judged. In these situations a judging panel will be convened, all of whom will read or study the works of art or literature and who in the end, although they may have widely differing views, will be able to agree on a particular work which may be the winner. There are a collection of complexities to the way that these situations are judged. It may perhaps be true that the piece that wins is no one person's favourite. My favourite may be as unacceptable to you as your favourite is to me but we may be able to achieve a consensus and agree that the one that is your and my second choice should be the winner as it is equally acceptable to both of us. These complexities need not concern us here. All we need to note is that there are situations in which, despite there being no objective standard, a clear decision can be made about which work of art is – objectively, in the sense in which the decision is made through an objective process – the best. Will this model work to identify the best interests of children in an objective fashion?

The answer to this must be ‘no’. First, it seems too trivial. The decisions that will be made on the basis of a child's objective best interests are on a completely different level to selecting the winner for the Booker or Turner prize. Secondly, in a book judging competition at least one approach will be that the panel agrees upon a set of rules. The rules may be agreed upon in a variety of ways, and perhaps there will be a scoring system with scores in different realms – imagination, originality, writing, plot – all scored and added, or a vote with the majority winning. However, in determining the best interests of a child, the judge decides. And it may be that those who disagree with the judgement can see no clear way that the judge has moved from the facts and the evidence of the case to judgement of the best interests of a child. Although the approach used in court requires that a clear list of benefits and harms is listed for each course, there is no clear calculus to establish the sum total of benefits and disbenefits for the child. The process which may be agreed in judging contests of art may be absent in the published reports of the court proceedings. Put another way, those reading a case may follow the reasoning and clear description of the evidence right up to the point where the judge decides, where the leap that the judge makes from fact and argument to judgement is incomprehensible. Thirdly, it is important to recognize that panels are convened for the Booker or Turner prize but, at least in the lower courts, only one judge is involved. If we accept that there are no objective best interests and so in assessing best interests people bring their own prejudices and particular conceptions of best interests, assessed from their own particular perspective, it is likely that the more people, of the appropriate experience or training, who are involved in coming to a decision the more likely it is that the group as a whole will have seriously engaged with the different perspectives. Or the larger the group, the more likely that group is to make a decision which fits with the consensus in society. Bridgeman herself describes the way that judges ‘… bring their own values, experiences and perspectives to their judging’.^12^ This makes it likely that when judging best interests judges will view best interests from one particular standpoint; they will have a narrower conception of best interests than a more broadly selected group would have. And if we seek objectivity in the process then bias will be less, or prejudices are more likely to be balanced, when those who judge best interests have a more diverse origin. Perhaps here we should note that when medical decisions are made for critically ill children they will be made by multidisciplinary teams which will usually include more than one representative of several different professional groups including doctors of different disciplines, nurses and physiotherapists. These professionals with different perspectives will bring a broader conception of interests than a single judge. Furthermore, they will all have interacted with the child. Fourthly, consensus is not achieved; there is a winner and one or more losers. I have described above the trade-offs that may be made in judging a book contest, but these negotiations will not take place in a court.

My claim is that objective best interests do not exist. If the response is that we have an objective approach to locate someone's best interests, to inject the *objectivity* into objective best interests, then this should be stated explicitly. And if this claim is made, we should ask what an objective process to decide on what we treat as an individual's best interests would be, and having answered that question we should implement whatever approach it recommends. I have described above some of the ways in which the court's decisions may fall short.^13^ In contrast, Bridgeman denies that there is an objective sense of best interests but argues that the courts can locate a child's best interests. Imagine that Mr Justice Holman and I have both painted or drawn a picture of a unicorn. If we both agree that unicorns do not exist, then it does not make sense to claim that the judge's drawing of a unicorn is a more realistic or more objective picture of a unicorn than my painting of a unicorn. Neither of us can claim a true picture of a unicorn. They are merely representations of something fictitious. And if we are explicit about this, then, with regard to best interests, it is likely that we can achieve a better understanding of the child's interests and the ways in which the notion of best interests may be distorted by a particular perspective. Towards the end of her paper, Bridgeman claims that ‘I argue for a conceptual framework of relational responsibilities in which decisions about the best interests of the child are informed by careful consideration of the child as an individual, of the responsibilities of parents and professionals arising from the distinct and different relationships they have with the child and the wider context which informs judgments about the future of critically-ill children such as the limitations of medical science, discriminatory attitudes and support available to those who care. Such an approach, I argue, would ensure a fuller assessment of best interests’.^14^ This describes a complete and full assessment of a child's interests, although we must be careful to concentrate on the *child's* interests, which must be appropriate. However, regardless of how thoroughly the child's interests are assessed, this is still not an objective notion of best interests. If there are no objective best interests, redeveloping a way of assessing them as relational responsibilities will not instil the objectivity into interests. And, to continue with my analogy, if there is no such thing as objective best interests, then everybody's portraits of unicorns are fictions.

To demonstrate the confusion that exists, consider Mr Justice Hedley's comments, when ruling on further treatment for Charlotte Wyatt ‘the concept of “intolerable to that child” … is a valuable guide in the search for best interests in this kind of case’.^15^ There are different approaches to the way that interests may be grounded; for example, some people believe that an objective list may be made of what a person's interests are. An alternative conception is that the best way to assess what is in someone's interests is what makes her most happy or least unhappy, or what fulfils her desires. These approaches are well described in the biomedical ethics arena by de Grazia.^16^ de Grazia argues that there is no agreement on a framework that could be used to assess best interests, but that the way one sees one's own life through one's own eyes is usually a significant component. If this is so, then an intolerable life must be so far from best interests as to be entirely unhelpful. Or if it is thought to be helpful, then a very strong bias towards the preservation of life has distorted the assessment of the child's interests a long way from best interests.

The confusion over interests towards the end of a child's life is not just an academic concern. Individuals in the population have very different views on what would be in a child's interests approaching the end of life. For example, recently a 13-year-old girl decided that she would refuse a heart transplant needed as a consequence of side-effects of treatment for cancer.^17^ Her parents supported her decision. The health-care team considered making the child a ward of court to oblige her to accept a transplant. The views expressed in online comment sites reflect the broad disagreement as to what would be the best course for the child, ranging from ‘Who in God's name do the council think they are!!! This is a very difficult time for the family, they need support not interference. The stupidity of the council is of astronomical proportions’. to ‘i am on my third draft now … i wish you would try some more Hannah as life is so important.’.^17^ This does not demonstrate that there is no objective concept of best interests, but indicates the wide disagreement about what would be in someone's interests.

One particular problem with interests in an intimate family is that of balancing the interests of family members. There must, as in political philosophy, be some way in which all the family members' interests are considered together as it will not be possible to act in each individual family member's best interests. Importantly, it is not possible to separate the interests of individuals within an intimate family. Parents have a very strong interest in the wellbeing of their child. For most parents, that their child or children should do well is among the most important of the parents' interests. The child's interests are irretrievably intertwined not only with that of the parents, but also other family members. That children should predecease their parents is against some of the parents' fundamental or overriding interests. Here the concern is that parents' interests in their child may lead them to demand that treatment should continue long past the point that there is any prospect that their child can recover, or long past the time when a competent adult would have been allowed to choose palliative treatment. Unpleasant treatment (the treatment of MB was described as cruel) may persist without the prospect that the child may survive at the request or on the demand of the child's parents.^18^ This must have been among the concerns in *An NHS Trust v MB* where the medical team supported a change of approach from aggressive, life-prolonging, treatment to palliative care, as did the guardian appointed on behalf of the child. Only the parents' application supported continuation of treatment. We must be sure that the parents' interests in the child do not override the child's interests, if we are to act in the child's best interests. Or we must be sure that parents', or perhaps in other cases the doctors',^19^ insistence on treatment is at least not too harmful to the child. This is difficult because the situation that a child should die with the parents feeling that ‘something else could be done’, and something could always be done, although that something may be painful and have a vanishingly small prospect of success,^20^ is a situation that should be avoided. The parents' memories of their child may be tarnished with the thought that things should have been different around the time of the child's death. But how far should this concern be allowed to push treatment in a way that is not in the child's interests?

I have thus argued that we have neither a clear conception of best interests, nor an objective approach to locate best interests. I have concentrated on a critical analysis of the legal notion of best interests to demonstrate that the legal conception falls short of objectivity. In this analysis I have argued that Bridgeman cannot maintain the two linked claims; first, that there are no objective best interests and, secondly, that the court can determine the child's objective best interests, as is their stated aim.

## Keown's claim

A second important area of disagreement is where Bridgeman uses Keown's response to Lord Mustill's unease in considering Tony Bland's interests, noting ‘We do not need to rely upon the interests of Anna's parents as her remaining interests, nor go so far as John Keown who, in his commentary on the Tony Bland case, asked “would it not have been contrary to his interests to use him as, for example, a sideboard?” to consider that critically-ill children do have interests'.^21^ It is right not to go as far as Keown. His paragraph in full is:

But could it be beneficial to feed and care for Bland even though he could not appreciate it? It is, however, perfectly possible to benefit someone, even if they are unaware of it, as where A, unbeknown to B, deposits a large amount in B's bank account, or speaks well of him to C. And to state, as did Lord Mustill, that Bland had ‘no best interests of any kind’ is, with respect surely false. Would it not have been contrary to his interests to use him as, for example, a sideboard?^22^

Keown (the questioner) assumes that Tony Bland has interests. What I and Lord Mustill ask is whether Tony Bland has interests. Keown does not argue for his position, he begs the question. To assume that he does have interests side-steps the question of whether he *does* have interests. However, Keown's example is well chosen. The credit of a large amount of money to Tony Bland's account would not have benefited him. He would not have known he was rich. He could not have chosen to use the money to pursue some long-held life goal. He could not have improved his life in his eyes in any way at all. Perhaps a response is that the money could have improved Tony's life in some way; a more comfortable bed or better wheelchair and so it *is* in his interests. But in making this response we use the word ‘interests’ in a particular way. There are two senses of the word interests – objective and subjective. For example, we could say that it is in a plant's interests that it receives regular watering and a judicious amount of sun. Or there may be a particular way to treat a dog that ensures that it grows well, has a wet nose and a glossy coat. This would be in the dog's interests. This notion of interests is what I call objective interests; this approach is objectively good for something or somebody. There is a subjective sense of interests. I, as an autonomous being, can choose my own interests; for example, I can choose to learn to paint instead of playing football, and you would be wrong to stop me regardless of the fact that objectively I am good at football and lousy at painting and despite the fact that it would be objectively better for all concerned, me included, if I stuck to football instead of imposing my art on others. It is this subjective sense of interests that is important and which is objected to when the claim ‘you're being paternalistic’ is made and we must recognize that the objective interests of plants or animals or persons are less important. It is the first, objective sense of interests that is in use when talking about Tony Bland's interests. Persons choose what is in their own interests, but this is not the way in which we could act in Tony Bland's interests; it is others choosing for him. This is the objective sense of interests that has less moral importance.

To continue with Keown's other example, I agree with him in one sense; it would be wrong to use Tony Bland as a sideboard. But this need not be because it is against his interests, if he has none, which is what is in dispute here. In an analogy, I think that there is no sense in which anyone would claim that *Mona Lisa* has interests. She, or it, is not the sort of thing that can have interests. Nevertheless, it would be wrong to cut *Mona Lisa* from her frame and use her as a tablecloth, but it would not be against *Mona Lisa*'s interests; she has none. So merely because it would be wrong to use *Mona Lisa* as a table cloth does not mean that it is against *Mona Lisa'*s interests. In the same way, although it would be wrong to use Tony Bland as a sideboard, this can be for reasons other than that it would be against his interests; that it offends against dignity, or respect or will offend others, for example.

## Conclusions

I have argued that there are particular problems if we claim to be able to decide on a child's *objective* best interests. Bridgeman responded taking the perspective of a legal academic. However, legal conceptions of best interests must be subject to criticism. My arguments demonstrate that her defence of the courts' ability to find an objective sense of children's best interests, while recognizing that there are no objective best interests, is unsuccessful. Unless we have a clearer understanding of interests, the justification that we are acting in the objective best interests of critically ill children when making medical decisions around the time of their death cannot be sustained. Furthermore, I have demonstrated that Keown's argument that Tony Bland, or others like him, have interests does not succeed.
